# Kynurenine pathway metabolism following prenatal KMO inhibition and in *Mecp2*^+/−^ mice, using liquid chromatography-tandem mass spectrometry

**DOI:** 10.1016/j.neuint.2016.09.012

**Published:** 2016-11

**Authors:** Caroline M. Forrest, Peter G.E. Kennedy, Jean Rodgers, R. Neil Dalton, Charles Turner, L. Gail Darlington, Stuart R. Cobb, Trevor W. Stone

**Affiliations:** a Institute of Neuroscience and Psychology, College of Medical, Veterinary and Life Sciences, University of Glasgow, Glasgow, G12 8QQ, UK; b Institute of Infection, Inflammation and Immunity, College of Medical, Veterinary and Life Sciences, University of Glasgow, Glasgow, G12 8QQ, UK; c Institute of Biodiversity, Animal Health and Comparative Medicine, College of Medical, Veterinary and Life Sciences, University of Glasgow, Glasgow, G61 1QH, UK; d WellChild Laboratory, Evelina London Children's Hospital, King's College London, Lambeth Palace Road, London, SE1 7EH, UK; e Department of Internal Medicine, Ashtead Hospital, Ashtead, Surrey, KT21 2SB, UK

**Keywords:** Kynurenine, Kynurenic acid, Quinolinic acid, Tryptophan metabolism, Rett syndrome

## Abstract

To quantify the full range of tryptophan metabolites along the kynurenine pathway, a liquid chromatography – tandem mass spectrometry method was developed and used to analyse brain extracts of rodents treated with the kynurenine-3-mono-oxygenase (KMO) inhibitor Ro61-8048 during pregnancy. There were significant increases in the levels of kynurenine, kynurenic acid, anthranilic acid and 3-hydroxy-kynurenine (3-HK) in the maternal brain after 5 h but not 24 h, while the embryos exhibited high levels of kynurenine, kynurenic acid and anthranilic acid after 5 h which were maintained at 24 h post-treatment. At 24 h there was also a strong trend to an increase in quinolinic acid levels (P = 0.055). No significant changes were observed in any of the other kynurenine metabolites. The results confirm the marked increase in the accumulation of some neuroactive kynurenines when KMO is inhibited, and re-emphasise the potential importance of changes in anthranilic acid. The prolonged duration of metabolite accumulation in the embryo brains indicates a trapping of compounds within the embryonic CNS independently of maternal levels. When brains were examined from young mice heterozygous for the meCP2 gene – a potential model for Rett syndrome - no differences were noted from control mice, suggesting that the proposed roles for kynurenines in autism spectrum disorder are not relevant to Rett syndrome, supporting its recognition as a distinct, independent, condition.

## Highlights

• Pregnant rats were treated with an inhibitor of kynurenine-3-monoxygenase.
• Levels of several kynurenine metabolites increased in the maternal and foetal brains.
• The maternal changes at 5 h disappeared by 24 h, but were maintained in embryos.
• No changes were noted in the brains of *Mecp2*^+/−^ mice.
• KMO inhibition but not *Mecp2*^+/−^ suppression alters kynurenine metabolism.

## 1 Introduction

The kynurenine pathway is the major route for tryptophan metabolism, oxidising around 95% of free tryptophan to several compounds with biological activity at glutamate receptors, such as quinolinic acid (Stone and Perkins, 1981, Stone and Darlington, 2002) and kynurenic acid (Perkins and Stone, 1982). Both these compounds have important actions on the *N*-methyl-d-aspartate (NMDA) sensitive population of receptors for glutamate at which quinolinic acid is an agonist and kynurenic acid is an antagonist, acting at the glycine/d-serine site of NMDA receptors (Birch et al., 1988).

Concentrations of kynurenate are altered in the brains of patients with disorders such as schizophrenia (Nilsson et al., 2005, Schwarcz et al., 2001; see Stone and Darlington, 2013) and there is strong evidence for a role also in Huntington's disease (Forrest et al., 2010, Schwarcz et al., 2009). Both of these disorders involve abnormalities in brain development or maturation and NMDA receptors are intimately involved in the early formation of the embryonic brain (Iwasato et al., 2000, Ramoa et al., 2001), where they influence progenitor migration, axon guidance, contact formation, dendritic and spine formation (Alvarez et al., 2007, Colonnese et al., 2005, Rajan and Cline, 1998, Ultanir et al., 2007). Increasing the endogenous concentration of kynurenic acid in the CNS would, therefore, be predicted to modify brain development.

Attempts to clarify the mechanisms by which the kynurenine pathway influences these aspects of brain function have involved inducing changes in the brain concentrations of kynurenines by the administration of kynurenine (Chess et al., 2007, Pocivavsek et al., 2012), pharmacological inhibition of the key kynurenine metabolic enzyme kynurenine-3-mono-oxygenase (KMO) (Forrest et al., 2013a, Forrest et al., 2013b, Khalil et al., 2014) or deletion of the gene for this enzyme in mice (Giorgini et al., 2013). Inhibition or deletion of KMO blocks the oxidation of kynurenine to 3-hydroxy-kynurenine, providing a higher concentration of kynurenine as a substrate for kynurenine aminotransferase, which metabolises it to kynurenic acid. These procedures have demonstrated that during early brain formation, *in utero*, an inhibitor of KMO produces changes in the morphological, biochemical and electrophysiological development of the brains of the offspring (Forrest et al., 2013a, Forrest et al., 2013b, Khalil et al., 2014, Pisar et al., 2014), indicating that activity along the kynurenine pathway is actively involved in early brain formation and its subsequent maturation.

As part of these latter studies, tissue samples were analysed by HPLC to determine the levels of tryptophan and its major metabolites in the embryonic and maternal brain 5 h and 24 h after the administration of the KMO inhibitor Ro61-8048 (Forrest et al., 2013a). However, samples of brain were also retained from the animals used in those studies with the aim of developing a highly sensitive mass spectrometry assay with which to examine changes simultaneously in the levels of all of the major kynurenine metabolites. This report presents the new data on brain levels of tryptophan, kynurenine, kynurenic acid, quinolinic acid, anthranilic acid, xanthurenic acid and 3-hydroxykynurenine, which support and extend our earlier observations. The question being addressed in the first part of the study is how KMO inhibition during gestation affects kynurenine metabolism in the maternal and embryonic brain and whether changes in the embryo could underlie the emergence of behavioural disorders postnatally.

In addition we have applied the new analysis system to examine kynurenine metabolites in the brains of female mice heterozygous for the X-linked *Mecp2* gene. Deletion of this gene has been considered to represent an accurate genetic model of Rett syndrome, a neurodevelopmental disorder affecting females and characterised by motor, cognitive and autonomic impairment as well as autistic features during a childhood regression phase (Chen et al., 2001, Guy et al., 2001). Riederer et al. (1986) reported increased levels of kynurenine in post-mortem brain tissue of patients suffering from Rett syndrome but, to our knowledge, no measurements have been made of the full complement of tryptophan metabolites in an animal model of the disorder. The question being addressed here is whether any changes in kynurenine metabolism can be identified in a mouse model of Rett syndrome.

## 2 Materials and methods

The use of animals has been described in the original studies from which the present brain samples were taken (Forrest et al., 2013a, Forrest et al., 2013b, Forrest et al., 2015). The work was carried out according to the regulations of the Animals (Scientific Procedures) act 1986 of the UK, administered and monitored by the Home Office. Briefly, pregnant female Wistar rats were housed alone with free access to food and water. Kynurenine oxidation to 3-hydroxy-kynurenine (3-HK) was blocked by 3,4-dimethoxy-*N*-[4-(3-nitrophenyl)thiazol-2-yl]benzenesulphonamide (Ro61-8048) (Röver et al., 1997) which inhibits kynurenine-3-mono-oxygenase (KMO) and shifts the catabolism of kynurenine away from 3-HK and towards the NMDA receptor antagonist, kynurenic acid (Cozzi et al., 1999, Clark et al., 2005, Forrest et al., 2013a, Forrest et al., 2013b). A dose of 100 mg/kg (i.p.) was identified as one suitable for repeated administration to rodents (Clark et al., 2005, Rodgers et al., 2009) and this was injected to the pregnant rats on days E14, E16 and E 18 of pregnancy. Control animals were injected with the saline vehicle. Two time points were selected for analysis of the brains, 5 h and 24 h after the final administration of Ro61-8048, at which times the animals were killed by an overdose of anaesthetic (25% w/v urethane in water, 2.5 g/kg i.p.) followed by cervical dislocation.

Female *Mecp2*^+/−^ mice and the wild type litternate controls were used. The *Mecp2*^*tm1.1Bird*^ mice were originally provided as a kind gift from Professor Adrian Bird (University of Edinburgh) and maintained on a C57BL/6 background. Animals were maintained on 12-h light/dark cycles with free access to normal mouse food. Mice were genotyped as described previously (Guy et al., 2001).

### 2.1 Brain sample extraction

Samples were kept on ice throughout the extraction procedure. Mouse brains were prepared by homogenising in 5 vols (wt/vol) of HPLC grade methanol. Samples were homogenised for 30 s using a polytron (Fisher Scientific, Loughborough, UK) then centrifuged at 10,000 *g* for 10 min at 4 °C. The supernatants were collected and filtered in MicroSpin PVDF centrifuge tubes (Alltech, Carnforth, Lancashire, UK) using centrifugation at 10,000 *g* for 5 min at 4 °C. The filtrates were stored at −80 °C until mass spectrometric analysis.

### 2.2 Brain sample preparation

For the measurement of tryptophan and kynurenine, 10 μl of blank (deionised water), aqueous standards, and filtered brain extracts were mixed with 150 μl of stable isotope mix in methanol (5 μl 10 mM ^2^H_5_-tryptophan and 5 μl 0.64 mM ^2^H_6_-kynurenine sulphate in 20 ml methanol), vortex mixed and centrifuged for 5 min at 21,000*g*.

For the measurement of kynurenic acid, anthranilic acid, 3-hydroxykynurenine, 3-hydroxyanthranilic acid, xanthurenic acid, quinolinic acid, and picolinic acid, 150 μl of blank (deionised water), aqueous standards, and filtered brain extracts were mixed with 15 μl of the stable isotope mix in methanol (1:1:1, 2 μM ^2^H_5_-kynurenic acid, 5 μM ^13^C_3_,^15^N-quinolinic acid, 40 μM ^2^H_3_-picolinic acid), vortex mixed and centrifuged for 5 min at 21,000*g*. Supernatants were transferred to a 96-deep well polypropylene plate, sealing mat applied, and the plate placed in the autosampler cooled to 7.5 °C, awaiting injection.

### 2.3 Kynurenine metabolite analysis

Several groups have established the value of analytical systems based on mass spectrometry for components of the kynurenine pathway which represents the main route for the oxidation of tryptophan (Marcos et al., 2016, Galba et al., 2016, Orsatti et al., 2015, Schwieler et al., 2015, Savitz et al., 2015, Zhang et al., 2015, Meinitzer et al., 2014, Lesniak et al., 2013, Zheng et al., 2012, Moller et al., 2012, Wilson et al., 2012, Notarangelo et al., 2012, Owe-Young et al., 2008, Amirkhani et al., 2002, Boni et al., 1994, Smythe et al., 2002, Kita et al., 2002, Saito et al., 1993). These systems have been applied to the measurement of individual kynurenines in blood, plasma or cerebrospinal fluid as well as components of the kynurenine pathway within brain tissue. In the present work, tryptophan, kynurenine, and kynurenine metabolites were measured by liquid chromatography-tandem mass spectrometry using a CTC PAL HTS-XT, Agilent 1260 Infinity liquid chromatograph, and AB SCIEX 6500 QTRAP mass spectrometer (AB Sciex UK Ltd, Warrington, UK). All results were calculated in Analyst 1.6.

*Analytical chemicals and reagents:* Tryptophan, kynurenine, kynurenic acid, anthranilic acid, 3-hydroxykynurenine, 3-hydroxyanthranilic acid, xanthurenic acid, picolinic acid and quinolinic acid were obtained from Sigma Aldrich, Poole, Dorset UK. The stable isotope labelled internal standards were: ^2^H_5_-tryptophan (QMx Laboratories, Thaxted, UK), ^2^H_6_-kynurenine sulphate and ^2^H_5_-kynurenic acid (CK isotopes, Ibstock, UK), and ^2^H_3_-picolinic acid and ^13^C_3_,^15^N-quinolinic acid (LGC standards, Teddington, UK). Acetonitrile and methanol were obtained from Rathburn Chemicals Ltd., Walkerburn, UK and Fisher Scientific UK Ltd, Loughborough, UK, respectively.

#### 2.3.1 Analyte tuning and MRM optimisation

Standards of each analyte were used to automatically tune in multiple reaction monitoring (MRM) mode. Transition optimisations were performed manually. Xanthurenic acid was significantly more sensitive in negative ion mode but, with the chromatographic conditions used, there was massive matrix ion suppression. Consequently, xanthurenic acid was measured in positive ion mode.

#### 2.3.2 Liquid chromatography-tandem mass spectrometry

Analyte retention times on Chirobiotic-T columns tend to be matrix dependent, hence the critical requirement for the appropriate stable isotope internal standards. Consequently, where stable isotopes were unavailable, two separate transitions, where possible, were used to calculate and then confirm the result.

#### 2.3.3 Positive ion MRM mode acquisitions

Isocratic chromatography of tryptophan and kynurenine (5 μl injection volume; data acquisition time 6 min), and kynurenic acid, anthranilic acid, 3-hydroxykynurenine, 3-hydroxyanthranilic acid, and xanthurenic acid (10 μl injection volume; data acquisition time 7 min) was performed on a 5 μm ASTEC Chirobiotic –T, 10 cm × 2.1 mm i.d. column and guard column, using 1:1 acetonitrile/water with 0.025% formic acid at a flow rate of 200 μl/min. MSMS parameters: Curtain Gas: 40, CAD Gas: medium, Ion Source voltage: 5250 V, Gas temperature: 400 °C, Gas 1: 25, Gas 2: 25.

#### 2.3.4 Negative ion MRM mode acquisitions

Isocratic chromatography of quinolinic acid and picolinic acid (10 μl injection volume; data acquisition time 4 min) was performed on a 5 μm ASTEC Chirobiotic –T, 10 cm × 2.1 mm i.d. column and guard column, using 62.5% water/acetonitrile at a flow rate of 225 μl/min. MSMS parameters: Curtain Gas: 45, CAD Gas: medium, Ion Source voltage: −4500 V, Gas temperature: 400 °C, Gas 1: 30, Gas 2: 30 (see Table 1).

### 2.4 Data analysis and statistics

All data are expressed as the mean ± 1 S.E.M with n values cited in the text of Figure legends. Statistical comparisons were performed using two-tailed *t* tests between two sets of data or ANOVA followed by Dunnet's test for comparing datasets to a single control set, or the Bonferroni test using selected datasets when multiple comparisons were required. A probability value of P ≤ 0.05 was used as the criterion for statistical significance.

### 2.5 Sources of compounds

Sources of the compounds used for analysis are listed above in section 2.3. The KMO inhibitor Ro61-8048 was partly obtained as a gift from Dr. S. Roever, F. Hoffmann-La Roche Ltd., Basel, Switzerland, and partly purchased from Tocris chemicals (Bristol, UK).

## 3 Results

### 3.1 Ro61-8048 treatment

**Fig. 1:**
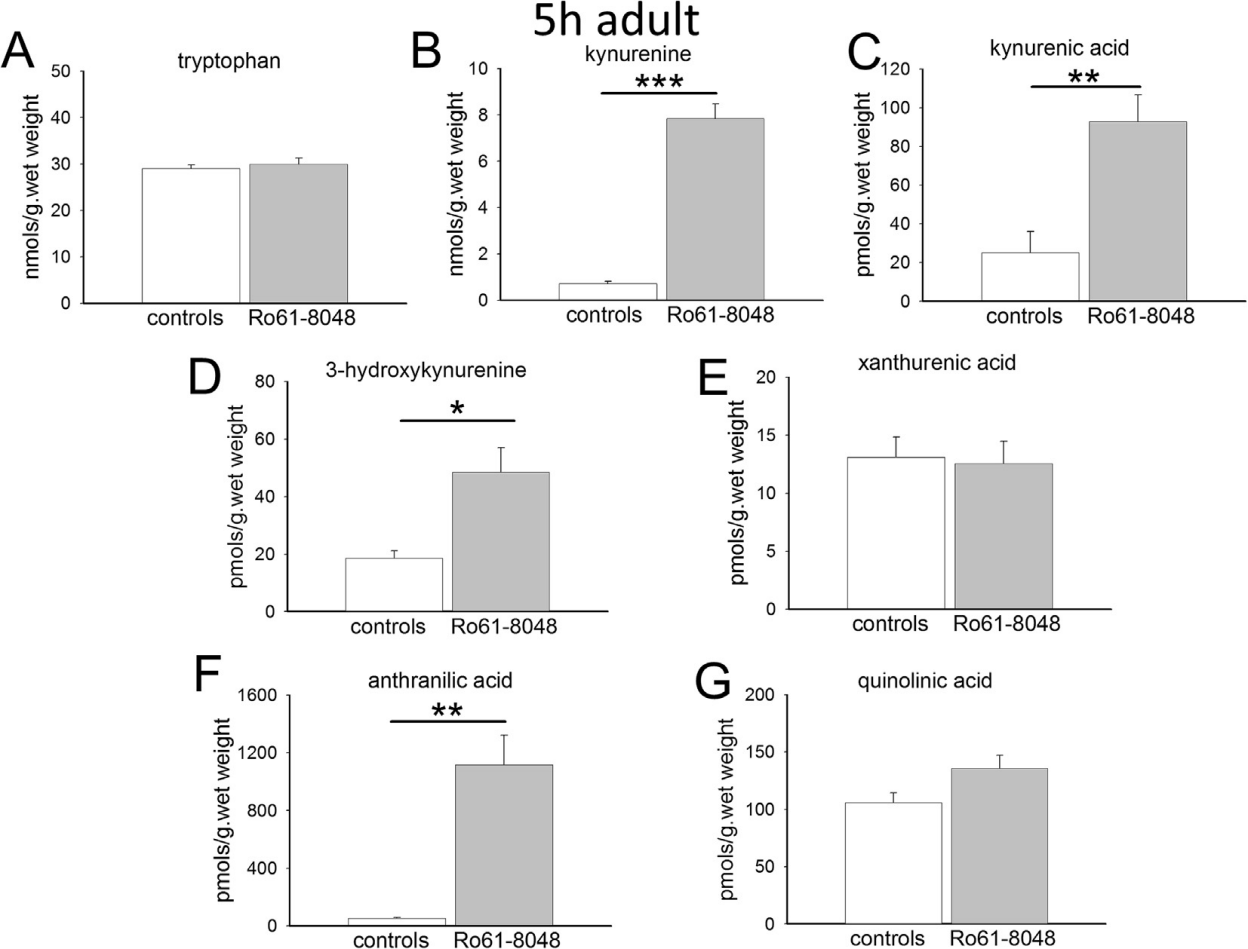
Tryptophan metabolism in adult female rat brains. The charts summarise the concentrations of (A) tryptophan, (B) kynurenine, (C) kynurenic acid, (D) 3-hydroxykynurenine, (E) xanthurenic acid, (F) anthranilic acid and (G) quinolinic acid in the brains of pregnant female rats 5 h after the intraperitoneal administration of Ro61-8048. *P < 0.05; **P < 0.01; ***P < 0.001 (paired *t*-test, n = 3).

**Fig. 2:**
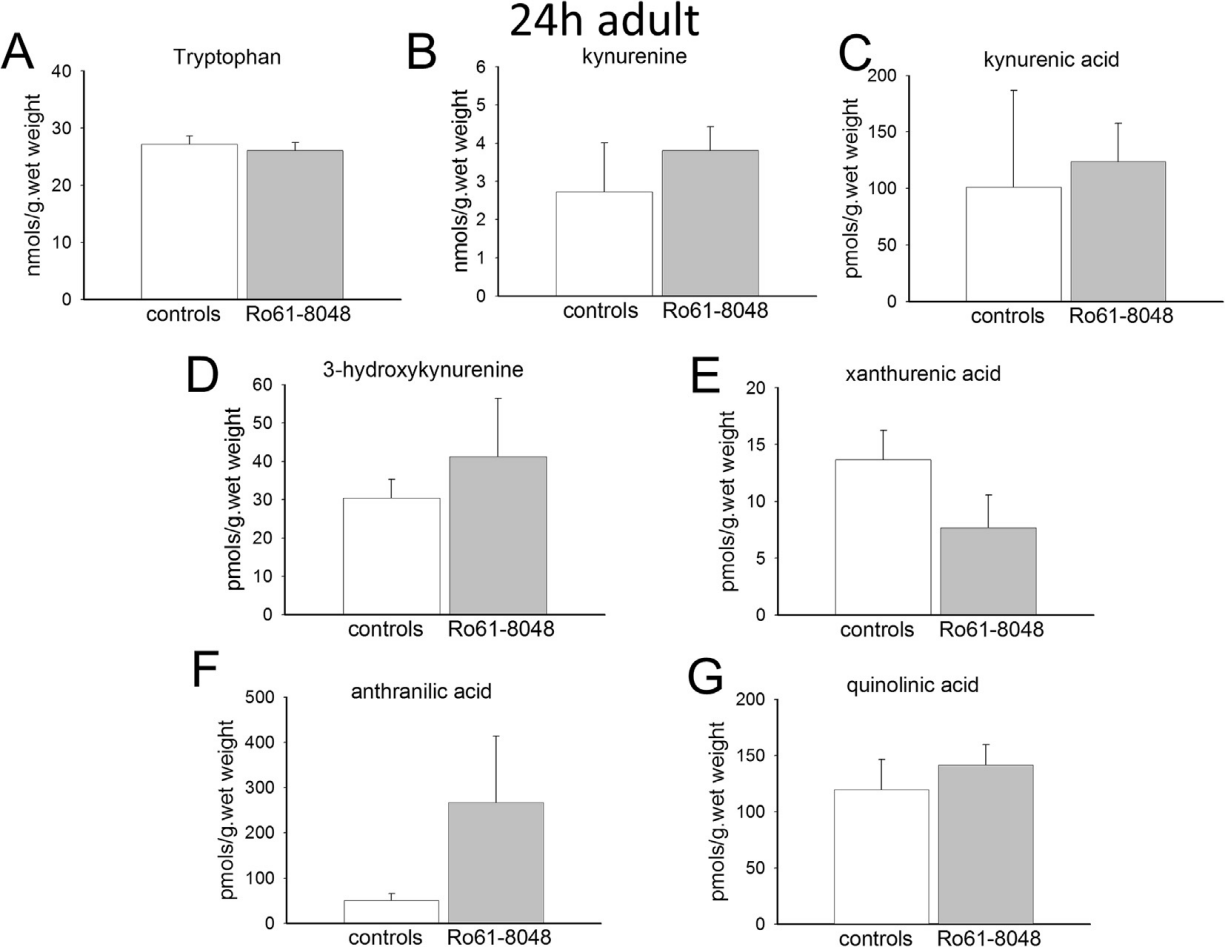
Tryptophan metabolism in adult female rat brains. The charts summarise the concentrations of (A) tryptophan, (B) kynurenine, (C) kynurenic acid, (D) 3-hydroxykynurenine, (E) xanthurenic acid, (F) anthranilic acid and (G) quinolinic acid in the brains of pregnant female rats 24 h after the intraperitoneal administration of Ro61-8048, a time at which there were no longer any significant differences (paired *t*-test, n = 3).

In the adult pregnant female, the administration of Ro61-8048 produced substantial and highly significant increases in the concentrations of the main kynurenine metabolites of tryptophan in the rat brain. Tryptophan itself showed no change after 5 h or 24 h, whereas kynurenine, 3-HK and kynurenic acid showed substantially increased levels after 5 h (Fig. 1). However, by 24 h following Ro61-8048 there were no significant differences between the control and treated animals (Fig. 2). There was also a significant rise in the concentration of anthranilic acid after 5 h which was no longer significant after 24 h partly due to a high variance (Fig 2). There was a strong trend for xanthurenic acid to be decreased by approximately 50% after 24 h but this was not significant (Fig. 2). Quinolinic acid levels did not change at either time point.

**Fig. 3:**
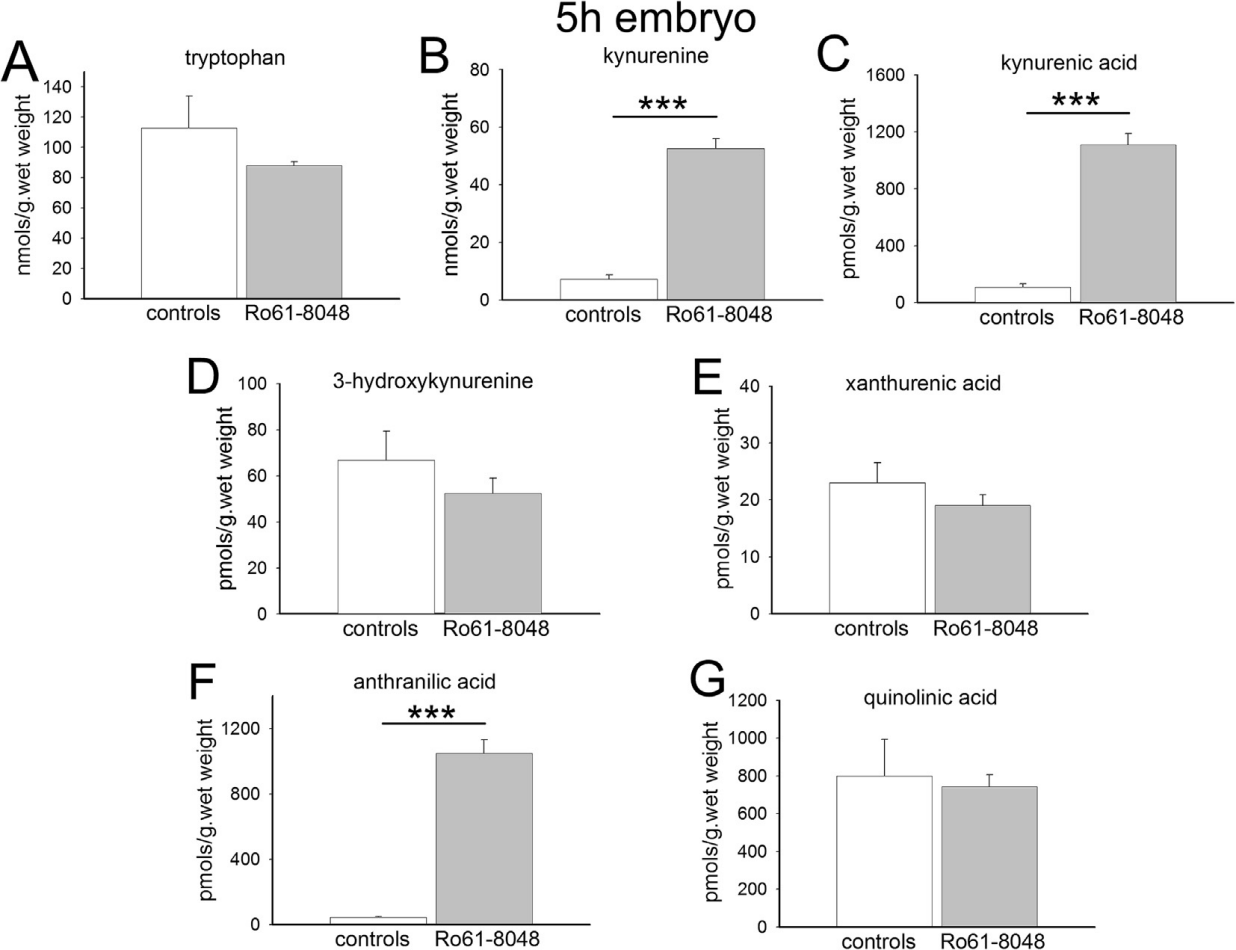
Tryptophan metabolism in embryo rat brains. The charts summarise the concentrations of (A) tryptophan, (B) kynurenine, (C) kynurenic acid, (D) 3-hydroxykynurenine, (E) xanthurenic acid, (F) anthranilic acid and (G) quinolinic acid in the brains of rat embryos 5 h after the intraperitoneal administration of Ro61-8048 to the mother. ***P < 0.001 (paired *t*-test, n = 6).

**Fig. 4:**
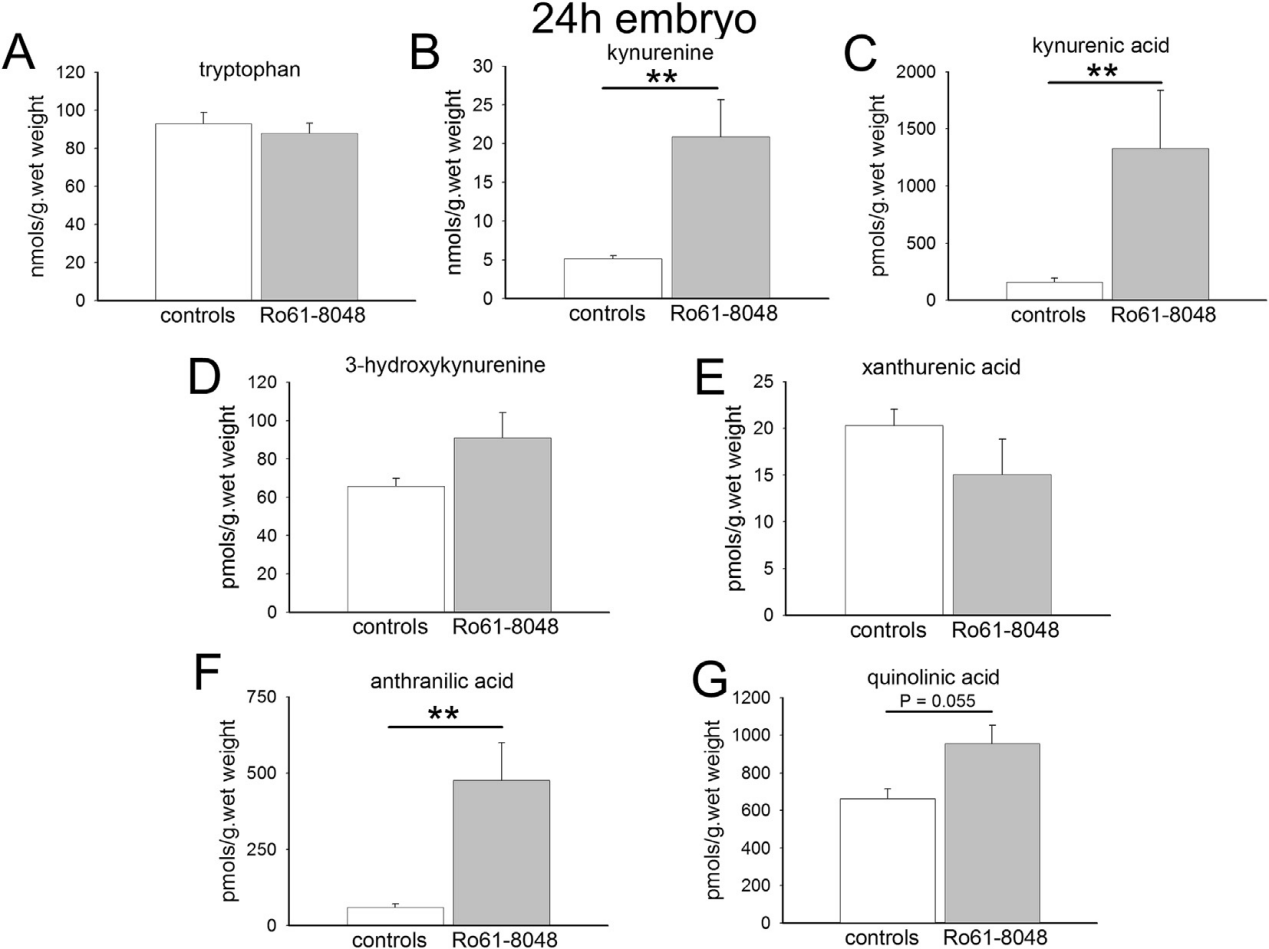
Tryptophan metabolism in embryo rat brains. The charts summarise the concentrations of (A) tryptophan, (B) kynurenine, (C) kynurenic acid, (D) 3-hydroxykynurenine, (E) xanthurenic acid, (F) anthranilic acid and (G) quinolinic acid in the brains of pregnant female rats 24 h after the intraperitoneal administration of Ro61-8048. **P < 0.01 (paired *t*-test, n = 6).

In the embryos, there were also highly significant increases in the concentrations of kynurenine, kynurenic acid and anthranilic acid at 5 h (Fig. 3), but these remained high, with little diminution at 24 h from the earlier levels (Fig. 4) in contrast to the adult data. There were no changes in the concentrations of 3-HK, xanthurenic acid or quinolinic acid at either time point.

The controls levels of kynurenine and kynurenic acid at 24 h are comparable in the adults (Fig. 2) and embryos (Fig. 4). The increased levels produced by Ro61-8048, however, remain around 10 times the control levels in the embryos (Fig. 4) whereas levels have returned to approximately normal in the adults (Fig. 2). There is a tendency for the levels of kynurenine and kynurenic acid to be lower in the adults at 5 h (Fig. 1) than at 24 h (Fig. 2). Since all the animals in this study were in the late stages of gestation, the difference may be related to the trauma of handling and injection, procedures which could have transiently depressed tryptophan metabolism for up to 5 h, returning towards normal by 24 h.

Concentrations of anthranilic acid were similar in the adult and embryo brains (around 50 pmol s/g. wet weight), but 3-HK levels were significantly higher in the embryos at 5 h (66.8 ± 12.6 pmol/g wet weight) and 24 h (65.68 ± 4.1 pmol/g wet weight) than in the maternal brains (18.65 ± 2.51, P = 0.02 and 30.35 ± 4.9 pmol/g wet weight, P = 0.005 respectively).

Interestingly, given the lack of effect of Ro61-8048 on overall levels of quinolinic acid, the concentration of this compound is around 6 times higher in embryo brains (Fig. 3, Fig. 4) compared with maternal brain (Fig. 1, Fig. 2).

### 3.2 Mecp2^+/−^ mice

**Fig. 5:**
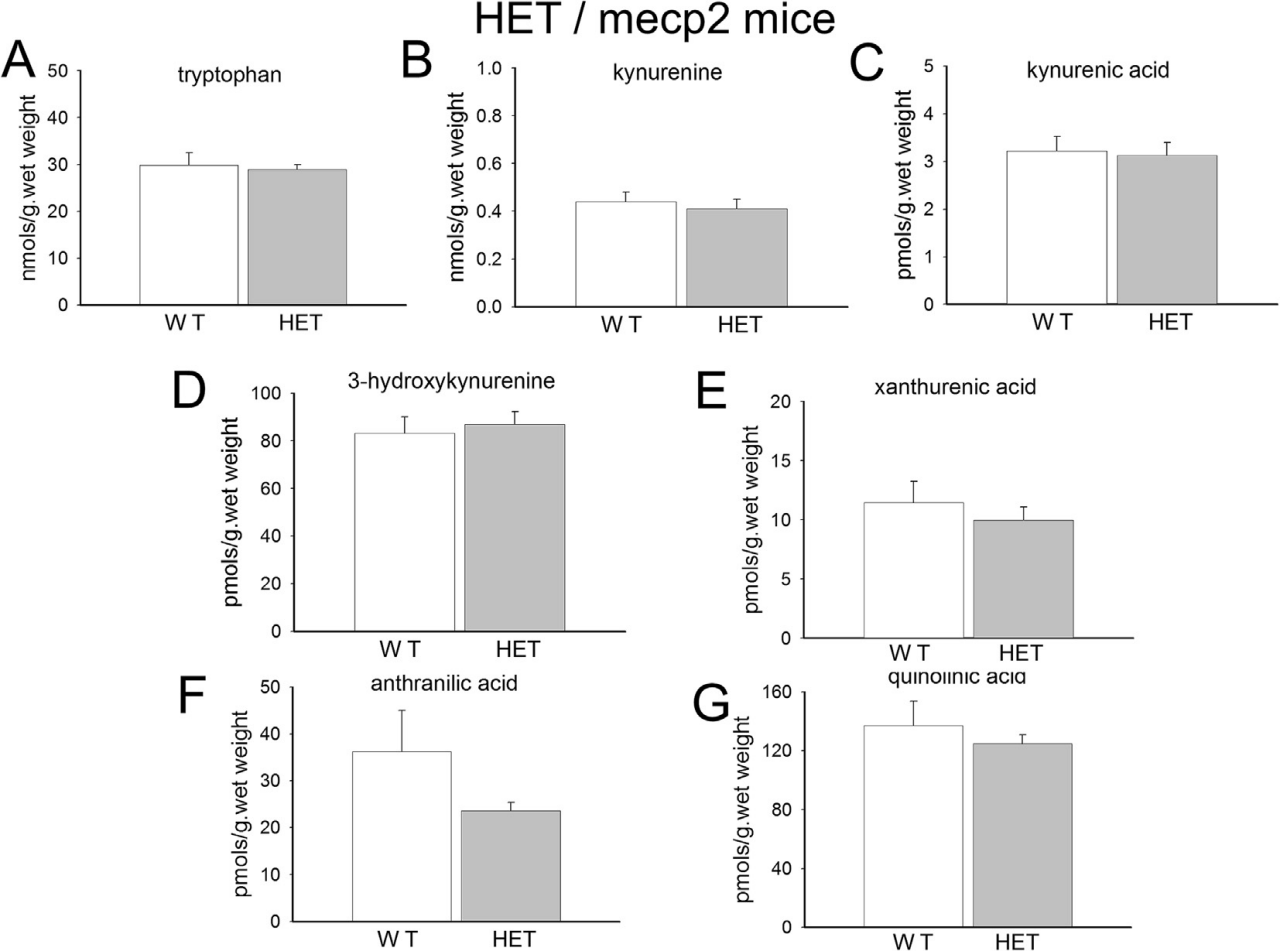
Tryptophan metabolism in the brains of *Mecp2*^+/−^ mice. The charts summarise the concentrations of (A) tryptophan, (B) kynurenine, (C) kynurenic acid, (D) 3-hydroxykynurenine, (E) xanthurenic acid, (F) anthranilic acid and (G) quinolinic acid in the brains of 3–6 week-old *Mecp2*^+/−^ mice. No significant differences were observed between the knockdown animals and their normal controls (paired *t*-test, n = 6).

In the brain samples from female mice (3–6 weeks old) mosaic for MeCP2 expression (lacking functional MeCP2 in ∼50% of brain cells as occurs in patients with Rett syndrome), no differences were detected in any of the metabolites examined including tryptophan, kynurenine, kynurenic acid, xanthurenic acid and quinolinic acid, all of which were completely unchanged compared with wild-type littermate control animals (Fig. 5). There was a tendency for anthranilate levels to be reduced in the mutants, but this did not achieve significance.

#### 3.2.1 Comparison of mouse and rat data

Although few direct comparisons have been made of all the brain tryptophan metabolite levels in rat and mouse brains, it was interesting to note that the basal levels of tryptophan (∼35 nmol/g.wet wt), kynurenine (∼0.4 nmol/g.wet wt), xanthurenic acid (∼12 pmol/g.wet wt), anthranilic acid (∼40 pmol/g.wet wt) and quinolinic acid (∼120 pmol/g.wet wt), were comparable in the mice and the untreated adult female rat controls described above. These values are also comparable with the baseline levels reported in previous studies (Tenorio-Lopez et al., 2007). The levels of kynurenic acid in mice, however, were only around 12% of those seen in rats (∼3 and ∼25 pmol/g.wet wt respectively) whereas the 3-HK levels were approximately 4-fold higher in the mice (∼80 pmol/g.wet wt) compared with the rat brains (∼20 pmol/g.wet wt). Compared with the embryonic rat brain, concentrations of tryptophan, kynurenine, kynurenic acid and quinolinic acid in the mouse brains were significantly lower, with kynurenic acid and quinolinic acid being of the order of 10-fold less in the mice.

Giorgini et al. (2013) employed the same background mouse strain (C57BL/6J) for the original conditional knockouts of KMO as were used for the *meCP2* knockout animals, although the KMO(−/−) animals were then back-crossed into an FVBN background. Nevertheless it was reassuring to note that the levels of tryptophan (∼30 nmol/g.wet wt), kynurenic acid (∼2.5 pmol/g.wet wt) and quinolinic acid (∼100 pmol/g.wet wt) were similar in the normal wild type animals in the two studies. However, kynurenine levels were around 10-fold less in the present study and anthranilic acid levels were 10-fold higher.

## 4 Discussion

The importance of understanding the manner in which kynurenic acid and quinolinic acid production are regulated lies in the recognition that their modulation of NMDA receptor function may be relevant to several CNS disorders. These include Huntington's disease (Forrest et al., 2010, Schwarcz et al., 2009) schizophrenia (Schwarcz et al., 2001, Nilsson et al., 2005; see Stone and Darlington, 2013) as well as ASD. Strong arguments have been advanced that these latter two conditions may be related to environmental influences on the early development of the brain *in utero* (Casanova, 2007). Given the actions of quinolinic acid as an NMDA receptor agonist and kynurenic acid as an antagonist, and that their concentrations are altered by infection (via interferon induction of cellular indoleamine-2,3-dioxygenase, IDO) and stress (via the corticosteroid induction of hepatic tryptophan-2,3-dioxygenase, TDO), it is reasonable to consider the possibility that changes in kynurenine metabolism could be involved.

The present results are broadly in agreement with data obtained using HPLC for kynurenine and kynurenic acid concentrations following KMO inhibition (Forrest et al., 2013a), which revealed large changes obtained 5 h after Ro61-8048 but which were restored to control levels after 24 h in the adult maternal brain while remaining elevated in the embryos. Increased synthesis of kynurenic acid has also been observed after Ro61-8048 treatment of mice in a study of the effects of cerebral malaria (Clark et al., 2005). In that study, kynurenate concentrations in the brain were increased up to 100-fold, while there was little change in the concentrations of quinolinic acid, again supporting the profile of metabolism obtained here.

The ability of tryptophan metabolism to change to this extent in the formation of kynurenine and kynurenic acid without any significant alteration in quinolinic acid synthesis remains unexplained. It has been proposed that the conversion of anthranilic acid to quinolinic acid could account for the observation, assuming that the quinolinate synthase activity necessary (Baran and Schwarcz, 1990) is regulated quite precisely in order to compensate for the loss of quinolinic acid normally generated from kynurenine via 3-HK. However, in the present analysis, concentrations of 3-HK were themselves elevated after 5 h in the adult brain, in parallel with the change in anthranilate levels, while quinolinic acid concentrations remained normal. Although it has not been demonstrated experimentally, it may be that there is a degree of inter-conversion between anthranilate and 3-HK which together are responsible for maintaining quinolinic acid synthesis in order to protect the generation of nicotinamide and nicotinamide adenine dinucleotide (NAD). It is unfortunate that the present analysis was unable to quantify the levels of 3-hydroxy-anthranilic acid, which is known to change in the opposite direction to anthranilate in some conditions, such as the presence of inflammation (Darlington et al., 2010).

It remains unclear whether Ro61-8048 is acting directly on this embryonic pathway. The blood-brain barrier is not fully functional until after birth, so that Ro61-8048 is likely to penetrate the embryonic brain far more readily than the maternal brain, resulting in the changes described here. However, the actual site of enzyme inhibition should not affect the overall results. Whether inhibition occurs within the embryo brain, within the maternal circulation or within the maternal brain, the consequence of enzyme inhibition should be, primarily, to increase the tissue levels of kynurenine. Since kynurenine is able to cross the placental and blood-brain barriers relatively easily, it should then lead to raised levels of kynurenic acid in all these tissues. The main uncertainty is that, in tissues where Ro61-8048 is present in very low concentration, kynurenine will still be converted to 3-HK and quinolinic acid at normal rates, rather than subnormal rates in the presence of KMO inhibition, so that the ratio of kynurenic acid to quinolinic acid may depend to some extent on Ro61-8048 penetration. This is potentially an important issue since it is the ratio between the levels of kynurenic acid (as a glutamate antagonist and neuroprotectant) and quinolinic acid (an NMDA receptor agonist and excitotoxin) that will determine the overall results of kynurenine pathway activity. The problem is likely to be moderated, however, by the fact that 3-HK, like kynurenine itself, can pass the placental and blood-brain barriers, thus compensating for any lack of tissue penetration by Ro61-8048.

However, the kynurenine profile seen here is not unique to the administration of a synthetic chemical (Ro61-8048). The deletion of the *kmo* gene in mice has been reported to yield a similar profile of tryptophan metabolism to that observed here, with substantially increased levels of kynurenine, kynurenic acid and anthranilic acid in the brain but relatively little change (approximately 20%, albeit statistically significant) in the presence of quinolinic acid (Giorgini et al., 2013). That knockdown study did, however, include measurements of metabolite levels in the plasma and liver, both of which did exhibit a significant decrease in quinolinic acid production. The constancy of quinolinate levels in the brain may, therefore, reflect the limited passage of ionised compounds across the blood-brain barrier, resulting in the entrapment of quinolinic acid within the tissue. This would support the growing belief that quinolinate concentrations should be regulated within strict limits within the brain if levels are not to be reached which would be neurotoxic (Guillemin, 2012, Guillemin et al., 2005, Stone et al., 2012).

### 4.1 Neurodevelopment

The ability of KMO inhibition to alter brain development supports the concept that the kynurenine pathway plays a key role in the early development of the CNS. The pathway is certainly present in the embryonic brain (Beal et al., 1992, Cannazza et al., 2001, Guillemin et al., 2001, Guillemin et al., 2005, Schwarcz and Pellicciari, 2002, Walker et al., 1999), with the potential ability to modify glutamate receptor activation via the balance between the agonist quinolinic acid and the antagonist kynurenic acid. The high level of quinolinic acid prenatally which we have reported was also noted by Cannazza et al., 2001).

A variety of external influences affect brain development, including maternal infection or stress (Hornig et al., 1999, Meyer and Feldon, 2010). The alterations of development are thought to underlie some neurological or psychiatric disorders in the offspring (Brown, 2006, Brown, 2011, Hornig et al., 1999, Meyer and Feldon, 2010). Interferons generated during infection, are potent inducers of indoleamine-2,3-dioxygenase and KMO (Carlin et al., 1989, Alberati-Giani et al., 1996, Silva et al., 2002, Brooks et al., 2016), and corticosteroids produced during stress activate TDO (Green and Curzon, 1975, Green et al., 1976, Nakamura et al., 1987, Young, 1981, Zunszain et al., 2012). These compounds could, therefore, mediate the effects of infection and stress on kynurenine metabolism.

Indeed any immune challenge to the mother or neonate which results, directly or indirectly, in the activation of central glia or peripheral macrophages, or changes in the levels of cytokines or kynurenines in the foetal or neonatal CNS, would alter that balance of concentrations and could seriously perturb neural development and plasticity (Zavitsanou et al., 2014). This could in turn increase the risk of CNS disorders, such as schizophrenia, Alzheimer's disease and Huntington's disease and would be consistent with evidence that genetic abnormalities of the kynurenine pathway are linked to disorders such as schizophrenia (Holtze et al., 2012, Sathyasaikumar et al., 2011, Stone and Darlington, 2013). The blockade of NMDA receptors by kynurenic acid produces neurochemical and behavioural changes which have been likened to those seen in schizophrenia (du Bois and Huang, 2007, Harris et al., 2003) and related to altered levels of kynurenic acid in patients as discussed above.

Overall, the present data strongly support the concept that constitutive activity of the kynurenine pathway during embryogenesis is intimately involved in early brain development. This conclusion has important implications for the converse possibility that external factors promoting or suppressing basal activity may interfere with brain development and, as a result, brain structure and function in the adult. Infection and stress activate the pathway and maternal exposure to these factors during pregnancy has been linked to the emergence of disorders such as schizophrenia in postnatal life. The kynurenine pathway could be a major contributor to that association.

### 4.2 Mecp2^+/−^ mice

Although Rett syndrome in now recognised and classified as a separate disorder, there are features of the syndrome during the regressive phase that closely resemble phenotypic aspects of ASD. Several groups have reported links between the kynurenine pathway and ASD including changes in the KMO gene (McFarlane et al., 2008) or in the levels of tryptophan, kynurenine, quinolinic or kynurenic acids in patients with ASD which could account for some symptoms of the condition via their actions on the nervous or immune systems (Boccuto et al., 2013, Casanova, 2007, Essa et al., 2013, McTighe et al., 2013, Schwartz, 2014, Zimmerman et al., 2005, Lim et al., 2016), while others have failed to find a relationship (Sweeten et al., 2006). A study which focussed on Rett syndrome concluded that the levels of kynurenine were increased in striatal regions of the post mortem human brain, whereas serotonin concentrations were reduced (Riederer et al., 1986). The authors concluded that the high kynurenine levels could be relevant to the seizures often experienced by patients with Rett syndrome as a result of the increased generation of the NMDA receptor agonist quinolinic acid (Stone and Perkins, 1981, Stone and Darlington, 2002).

The use of female *Mecp2*^+/−^ represents an accurate and sex specific model of Rett syndrome since it is only female children who develop recognisable symptoms typically survive into adulthood. The deletion results in far more severe difficulties in male children and is usually lethal in infancy. The absence of changes in tryptophan metabolites in the *Mecp2*^+/−^ mice might therefore indicate that changes in tryptophan metabolism along the kynurenine pathway are not altered in Rett syndrome without seizures. However, a mouse model with a single selected genetic mutation is not the same as a human being in which the effects of any genetic change have been partially compensated by behavioural adaptation or genetic modifiers. Disease phenotypes are typically less severe in mice and their onset more delayed. However, oxidative stress is known to precede the onset symptoms in mouse models of Rett syndrome (De Felice et al., 2014) and the fact that we did not observe significant alterations in kynurenine metabolism may suggest that this pathway is of lesser importance in the pathogenesis of Rett syndrome. It is also possible that the changes in kynurenine metabolism that have been described in ASD or Rett syndrome are secondary to the behavioural abnormalities, reflecting the complex physiological and psychological reaction to the primary cause in humans which would not be expected to be shared by mice. Viewed overall, the results might indicate that the reported changes in kynurenine metabolism should be considered in the category of epi-phenomena possibly related more to the stress experienced by sufferers of ASD or Rett syndrome or to co-incident infections and chronic low level inflammation.

The observation that kynurenine levels were lower in the wild type C57BL/6J mice here than in the study of kmo deletion by Giorgini et al. (2013), whereas anthranilic acid levels were around 10-fold higher, may reflect the different genomic background of the animals since the C57BL/6J mice used for the original conditional knockouts were subsequently back-crossed at least six times to an FVBN background, so that genomic features of the latter strain should predominate. Alternatively, the differences might indicate represent a gender difference since only female mice were used here, whereas Giorgini et al. (2013) do not specify gender and presumably used a mixed population.

In another respect, the use of these mice has served as a control of the new LC-MSMS system, since intrinsic variability in the analysis would be predicted to generate some spurious differences in metabolite concentrations. If the changes in tryptophan metabolism demonstrated above in KMO deficient animals are viewed as the positive control experiments, the failure to find differences between the *Mecp2*^+/−^ mice and their normal controls can be taken to support the reliability, reproducibility and robustness of this LC-MSMS system.

## Figures and Tables

**Table 1:** Values for ion MRM mode acquisitions.

	*m*/*z*	DP	EP	CE	CXP	ST1
*Positive ion MRM mode acquisitions*:						
Tryptophan	205.16/146.00	6	10	25	10	−13
^2^H_5_-tryptophan	210.16/151.00	6	10	25	10	−13
Kynurenine	209.10/94.10	71	10	21	12	−13
^2^H_6_-kynurenine	215.10/98.10	71	10	21	12	−13
Kynurenic acid	189.93/144.00	46	10	30	12	−13
^2^H_5_-kynurenic acid	194.93/149.00	46	10	30	12	−13
Anthranilic acid	138.20/92.00	41	10	29	12	−13
Anthranilic acid, confirmation	138.20/120.20	41	10	15	10	−13
3-hydroxykynurenine	225.10/110.10	61	10	23	14	−13
3-hydroxykynurenine, confirmation	225.10/208.20	33	10	14	10	−13
3-hydroxyanthranilic acid	154.10/80.10	35	10	36	15	−13
Xanthurenic acid	206.00/160.00	45	10	44	12	−13
*Negative ion MRM mode acquisitions:*						
Quinolinic acid	166.044/122.100	−30	−10	−14	−9	13
^13^C_3_,^15^N-quinolinic acid	170.044/126.100	−30	−10	−14	−9	13
Picolinic acid	121.989/77.800	−120	−10	−16	−31	13
^2^H_3_-picolinic acid	124.880/81.000	−30	−10	−15	−10	13

Abbreviations: Declustering Potential (DP), Entrance Potential (EP), Collision Energy (CE), Exit Potential (CXP), Prefilter (ST1).
